# DNA-PKcs Inhibition Sensitizes Cancer Cells to Carbon-Ion Irradiation via Telomere Capping Disruption

**DOI:** 10.1371/journal.pone.0072641

**Published:** 2013-08-27

**Authors:** Xin Zhou, Xin Zhang, Yi Xie, Kaoru Tanaka, Bing Wang, Hong Zhang

**Affiliations:** 1 Department of Heavy Ion Radiation Medicine, Institute of Modern Physics, Chinese Academy of Sciences, Lanzhou, China; 2 Key Laboratory of Heavy Ion Radiation Biology and Medicine of Chinese Academy of Sciences, Lanzhou, China; 3 Key Laboratory of Heavy Ion Radiation Medicine of Gansu Province, Lanzhou, China; 4 Radiation Risk Reduction Research Program, Research Center for Radiation Protection, National Institute of Radiological Sciences, Inage-ku, Chiba, Japan; The University of Hong Kong, China

## Abstract

Heavy-ion irradiation induces a higher frequency of DNA double strand breaks (DSBs) which must be properly repaired. Critical shortening of telomeres can trigger DNA damage responses such as DSBs. Telomeres are very sensitive to oxidative stress such as ionizing radiation. The DNA-dependent protein kinase catalytic subunit (DNA-PKcs) is the central component in the non-homologous end joining (NHEJ) repair complex and participates in telomere maintenance. Therefore, it is expected to enhance the cell killing effect of heavy-ion irradiation via DNA-PKcs inhibition. To test this hypothesis, cellular radiosensitivity was measured by the clonal genetic assay. DNA damage repair was relatively quantified by long PCR. Apoptosis was quantified by flow-cytometric analysis of annexin V/PI double staining, and senescence was analyzed by galactosidase activity. Telomere length was semi-quantified by real-time PCR. P53 and p21 expression was determined by western blotting. Our data demonstrated that MCF-7 and HeLa cells with DNA-PKcs inhibition were more susceptible to carbon-ion irradiation than Those without DNA-PKcs inhibition. Even though NHEJ was inhibited by the DNA-PKcs specific inhibitor, NU7026, most DNA damage induced by carbon-ion irradiation was repaired within 24 hours after irradiation in both cell lines. However, potential lethal damage repair (PLDR) could not restore cellular inactivation in DNA-PKcs inhibited cells. MCF-7 cells showed extensive senescence and accelerated telomere length reduction, while HeLa cells underwent significant apoptosis after irradiation with NU7026 incubation. In addition, both cell lines with shorter telomere were more susceptible to carbon-ion radiation. Our current data suggested that DNA-PKcs inhibition could enhance cellular sensitivity to carbon-ion radiation via disturbing its functional role in telomere end protection. The combination of DNA-PKcs inhibition and carbon-ion irradiation may be an efficient method of heavy-ion therapy.

## Introduction

Telomeres are specialized DNA-protein structures that cap the ends of chromosomes. About 90% of cancer cells contain short telomeres, but exhibit high telomerase activity. As cancer cells divide more often, they possess, on average, shorter telomeres than normal cells. Therefore, without proper telomerase function to maintain telomere length, telomeres in cancer cells can shorten at a faster pace than normal cells. Critically short telomeres can lead to chromosome aberrations [1] and induce the DNA damage response (DDR) [2]. Thus, telomere length may be an important factor for cancer cell inactivation.

Telomeres play important roles in cellular responses to DNA-damaging agents such as ionizing radiation (IR) [3]. Several lines of evidence have suggested that telomere maintenance is related to radiosensitivity. For instance, both the radiosensitive AT cells and ABS cells showed a number of telomere-associated defects and their radiosensitivity-related protein is present in telomeres [4]–[7]. Double strand breaks on telomeres must be properly processed by telomerase with the help of telomere binding proteins. If not, the breaks on telomeres could initiate chromosome degradation and/or rearrangements, DDR and eventually lead to cell death [8].

High LET radiation can induce greater and more complex DNA damage than low LET radiation. Low LET radiation mainly produces single-strand breaks (SSBs), whereas high LET radiation primarily produces DSBs and clustered damage, which represent a dangerous form of damage. If not properly repaired, DSBs cause genetic changes and/or cell death. The differential responses of cells following exposure to irradiation at different LET values may correspond to the fact that the nature of DNA damage is distinct. There are reports showing that telomere structure is particularly susceptible to oxidative stress [9]. Therefore, high LET radiation may cause more severe damage to telomeres than low LET radiation. As most cancer cells harbor shorter telomeres than normal cells, the telomeres in cancer cells may be more likely to decrease to a critical length with telomerase inhibition. Therefore, we postulated that the cell killing effect of heavy-ion irradiation could be enhanced by interference due to telomere elongation in cancer cells.

In this study, we showed that the radiosensitivity of MCF-7 and HeLa cells was greatly enhanced by NU7026, a DNA-PKcs specific inhibitor, following carbon-ion irradiation. Further investigations demonstrated that with a limited effect on DNA repair capacity, MCF-7 cells treated with NU7026 showed accelerated telomere loss after carbon-ion irradiation. As DNA-PKcs is not only the key component in the NHEJ repair complex, but is also engaged in telomere function, we postulated that NU7026 could enhance radiosensitivity by interfering with telomerase access to the telomere after carbon-ion irradiation.

## Materials and Methods

### Cell Culture and Irradiation Treatment

The human breast cancer cell lineMCF-7 and cervix cancer cell line HeLa were purchased from the American Type Culture Collection. Cells were maintained in Dulbecco’s Modified Eagle’s Medium (Gibco, USA) supplemented with 10% fetal bovine serum. Cells were cultured in 5% CO_2_ in humidified air at 37°C.

X-ray were generated with an X-ray machine (Pantak-320S, Shimadzu, Japan) operated at 200 kVp and 20 mA using a 0.5-mm Aluminum+0.5-mm copper filter. An exposure-rate meter (AE-1321 M, Applied Engineering Inc, Japan) was used for the dosimetry. The dose rates were 1.1 Gy/min. Cells in exponential growth were irradiated at room temperature, with non-irradiated culture cells (control) which were handled in parallel with the irradiated samples.

Carbon-ion irradiations were performed at room temperature at the Heavy Ion Medical Accelerator Center (HIMAC) of the National Institute of Radiological Sciences (Chiba, Japan), with 290 MeV/n carbon-ion; the LET value for carbon-ion was 40 KeV/µm. The dose rates were 1 Gy/min. Some irradiation procedure was also carried out in Heavy Ion Research Facility in Lanzhou HIRFL, Institute of Modern Physics, Chinese Academy of Sciences, Lanzhou, China with 300 MeV/n carbon-ion and a LET value of 49 KeV/µm.

### Clonogenic Assay

Cells in exponential growth were seeded in petri dishes for colony formation. 1000–10000 cells were seeded within one hour after irradiation based on the radiation dosage received. For the examination of potentially lethal damage recovery (PLDR), cells were cultured in the medium for a recovery period of 24 h after irradiation and then seeded. Cells were further incubated until the colonies formed were large enough to be counted, while still being separable. The colonies were then fixed with methanol and stained with 0.6% Giemsa solution. The colonies were counted manually. Only colonies containing more than about 50 cells were scored.

### Apoptosis Assay

Quantification of apoptotic cells was obtained using the Annexin V-FITC detection kit (beyotime, CN) according to the manufacturer’s protocol. Data were evaluated with Flowjo 7.2.1 software. Following controls were used to set up compensation and quadrants: unstained cells and cells stained with FITC-annexin V or with PI alone.

### Senescence Assay

MCF-7 cells were washed twice with PBS and fixed with 2% formaldehyde, 0.2% glutaraldehyde for 5 min. The cells were then washed again with PBS and stained with a solution of 1 mg/ml 5-bromo-4-chloro-3-inolyl-ß-galactosidase in dimethylformamide (20 mg/ml stock), 5 mM potassium ferrocyanide, 150 mM NaCl, 40 mM citric acid/sodium phosphate, pH 6.0, and 2 mM MgCl_2_. Following overnight incubation at 37°C, the cells were washed twice with PBS and then photographed.

### QPCR for Telomere Length Measurement

The FTC-3000 qPCR system (FUNGLYN, CA) was used for analysis. Total genomic DNA of 50 ng were used for telomere length assay, in a 20 µl reaction containing 1X SYBR Premix Ex Taq II (TaKaRa, Japan) and 200 nmol/l each primer. The sequence information regarding the primers was listed in Table 1. Triplicate reactions were performed for each marker using the following thermal cycling profile adapted from Cawthon [10]: 15 min at 95°C at stage1; 2 cycles of 15 s at 94°C, 15 s at 49°C at stage 2; and 32 cycles of 15 s at 94°C, 15 s at 58°C, 15 s at 72°C with signal acquisition at stage 3. The 72°C reads provided the Ct values for the amplification of the telomere and c-myc template. Relative quantification approach (△△Ct) was used according to method previously described [11]. Each test was carried out in triplicate.

**Table 1:** Primers for real-time PCR and long PCR.

Telomere	Sense: 5′-CGG TTT GTT TGG GTT TGG GTT TGG GTT TGG GTT TGG GTT-3
	Antisense: 5′-GGC TTG CCT TAC CCT TAC CCT TAC CCT TAC CCT TAC CCT-3′
C-Myc	Sense: 5′-TGG GAT TAC ACG TGT GAA CCA ACC-3′
	Antisense: 5′-GCT CTA CCC TCT CCT CTA CCG TCC-3′
HPRT	Sense: 5′-GAG GGC CAA GTT GGA CAG TG-3′
	Antisense: 5′-TTG CGG TTG TTG CTG ATC TG-3′

### DNA Damage Repair Assay

Long PCR for mtDNA damage evaluation was performed using the GeneAmp XL PCR kit (PerKin–Elmer, Boston, MA). Quantitative long PCR were performed in an Eppendorf Mastercycler PCR system (Eppendorf, Hamburg, Germany). The PCR cycle test was performed before to ensure the PCR in the exponential phase. The sequence information of the primers was listed in Table 1. The PCR was initiated with a 75°C hot-start addition of the polymerase and allowed to undergo the following profile: an initial denaturation for 1 min at 94°C followed by 25 cycles for large fragments or 20 cycles for small fragments of 94°C denaturation for 15 sec and 68°C extension for 15 min. A final extension at 72°C was performed for 10 min at the completion of the profile. An aliquot of each PCR product was resolved on a 1% vertical agarose gel and electrophoresed in TBE for 4 hr. The gels were then digitally photographed and quantified with FluorChem FC2 (Alpha Innotech corporation). The DNA damage was quantified by comparing the relative level of amplification of the large fragments of DNA (10.4-kb HPRT gene) normalizing this to the amplification of smaller (54-bp c-myc) fragments.

### Establishment of Cells with Shorter Telomere

Cells were incubated with 2 µM MST312 (Sigma, USA) for 50–70 days in Dulbecco’s Modified Eagle’s Medium (Gibco, USA) supplemented with 10% fetal bovine serum. Cells were cultured in 5% CO_2_ in humidified air at 37°C. The medium with 2 µM MST312 was replaced every 3 to 4 days. Telomere length was determined by QPCR as described above. MST312 was removed from the medium 24 hours before radiation procedure.

### Cytotoxicity Assay

Cytotoxicity of MST312 was determined by real-time monitoring of adherent cells by the RT-CES System (ACEA Biosciences, USA). 10000 cells were seeded in each 360 µL well with 200 µL medium before measurement. The medium was replaced every 3 days. Proliferation of MCF-7 and HeLa cells was recorded every 2 hours for 144 hours.

### Statistical Analysis

Statistical analysis was performed on the means of the data obtained from at least three independent experiments. Data are presented as means±SD. Student’s t-test program in Microsoft Excel was used to detect statistical significance. *p*<0.05 was considered to be statistically significant.

## Results

### NU7026 Treatment Enhanced Cellular Inactivation via Apoptosis and/or Senescence in Cancer Cells after Carbon-ion Irradiation

**Figure 1:**
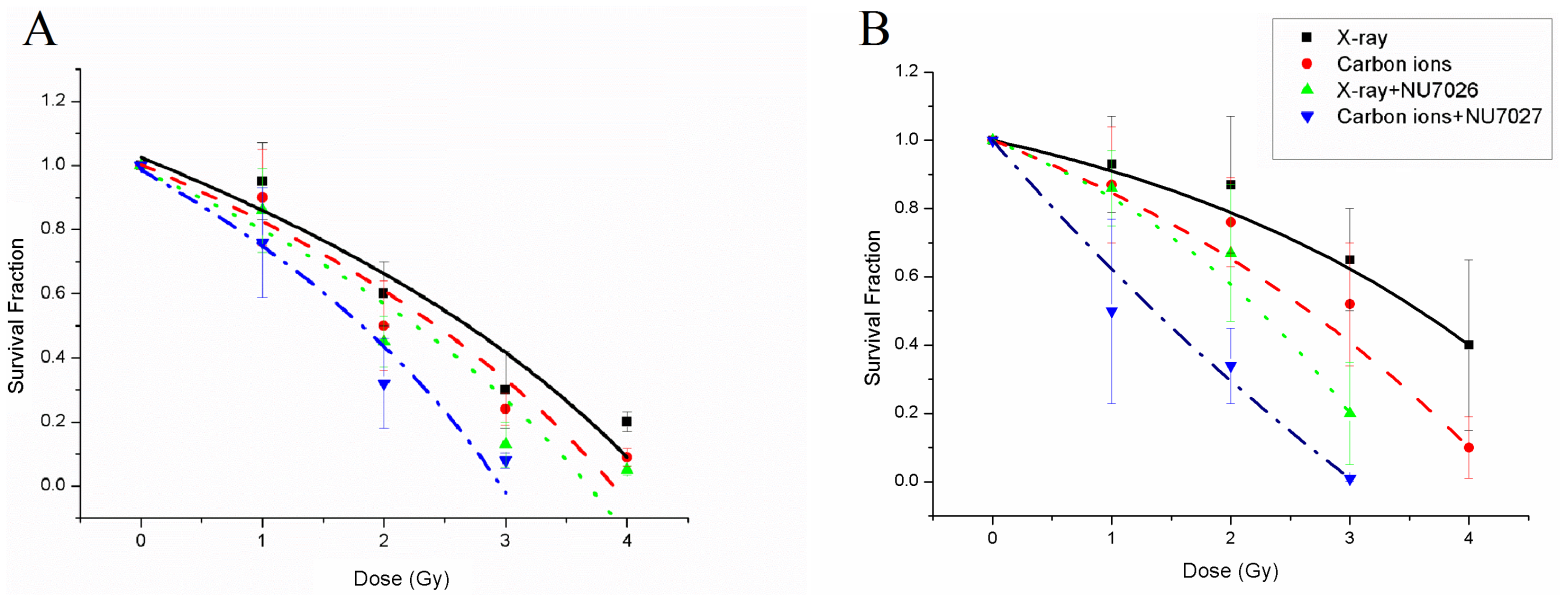
Effect of NU7026 on clonogenic survival of MCF-7 (A) and HeLa cells (B) after ionizing radiation. Cells were incubated with 10 µM NU7026 for 3 hours before irradiation.

**Figure 2:**
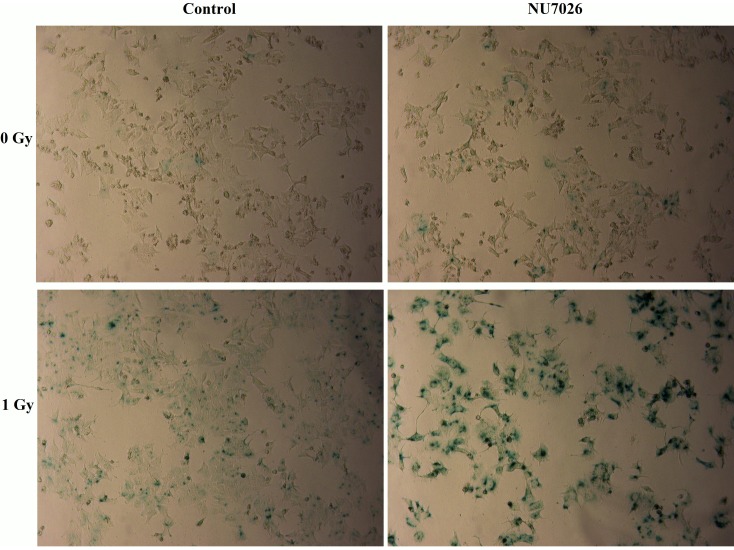
Cellular senescence of MCF-7 cells with/without 10 µM NU7026 treatment 30 days after 1 Gy carbon-ion irradiation.

The radiosensitivity of cells was examined by clonogenic assay. As illustrated in Figure 1, the radiosensitivity of NU7026-treated cells was greater than control in both cell lines. The cellular inactivation effect of carbon-ion irradiation was greater than that of X-rays. To further investigate cell fate after irradiation and/or NU7026 treatment, cellular apoptosis and senescence were determined by annexin/PI double staining and ß-galactosidase histochemical staining, respectively. Apoptosis in MCF-7 and HeLa cells was not significantly elevated when treated with carbon-ion irradiation or NU7026 alone, but was markedly increased by the combination of carbon-ion irradiation and NU7026 treatment (Table 2). MCF-7 cells treated with NU7026 after carbon-ion irradiation showed extensive cellular senescence 30 days after irradiation; carbon-ion irradiation alone also induced senescence, but to a much lesser extent (Figure 2). HeLa cells underwent significant population loss via apoptosis 48 hours after irradiation and cannot sustain proliferation afterwards.

**Table 2:** DNA-PKcs inhibition and telomere shortening led to increased apoptosis in MCF-7 and HeLa cells after carbon-ion irradiation.

	Early apoptotic cells		Late apoptotic/necrotic Cells	
	MCF-7	HeLa	MCF-7	HeLa
Control	0.06%±0.03	0.08%±0.02	0	0
NU7026	0.09%±0.03	0.08%±0.03	0.04%±0.03	0.03%±0.03
1 Gy Carbon-ion	0.06%±0.05	0.12%±0.06	0.03%±0.03	0.13%±0.04
NU7026+1 Gy Carbon-ion	8.25%±0.35* ^#^	12.25%±0.63* ^#^	1.54%±0.17* ^#^	30.95%±4.86* ^#^
Shorter telomere+1 Gy Carbon-ion		28.63%±3.59* ^#^		53.28±8.24* ^#^

Cells were incubated with 10 uM NU7026 for 3 hours before irradiation. 48 hours after 1 Gy carbon-ion irradiation, apoptosis was evaluated by Annexin V/PI double staining using FACS.

* p<0.01 versus control;

#p<0.01 versus 1 Gy carbon-ion irradiation.

### Enhanced Radiosensitivity was Independent of DNA Damage Repair Capacity

**Figure 3:**
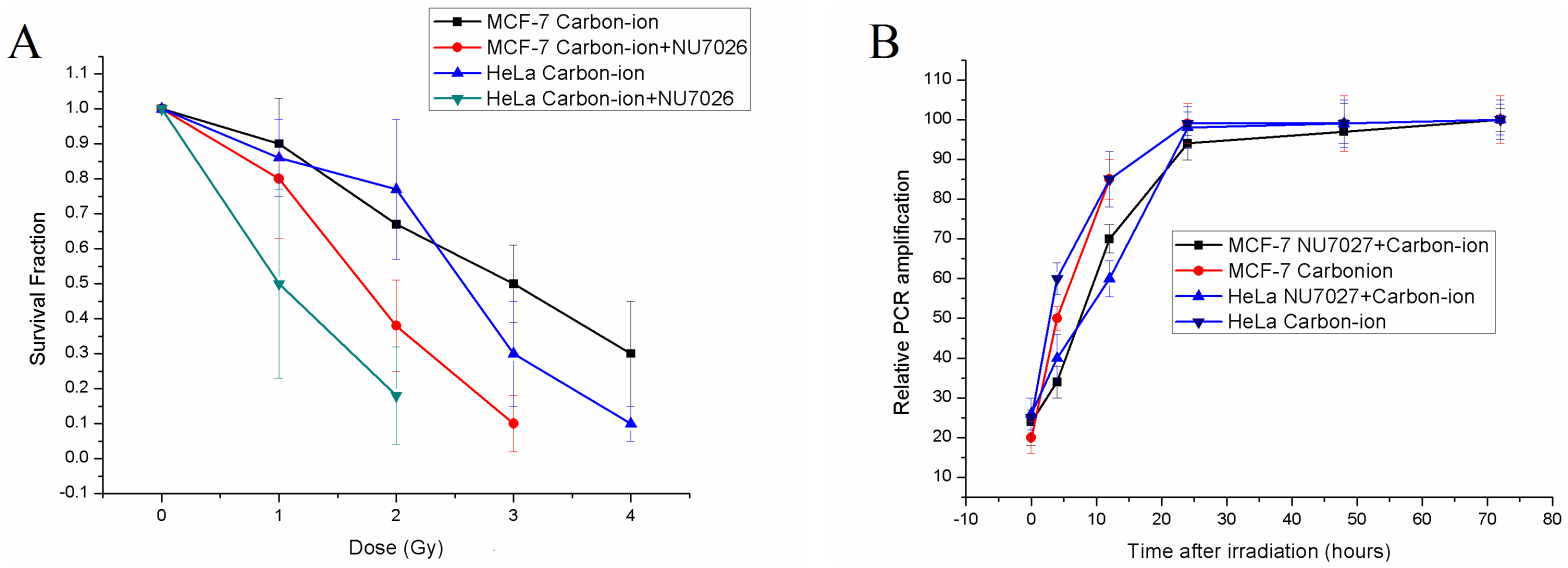
NU7026 enhanced radiosensitivity independent of its role in DNA damage repair. A. The survival fraction of irradiated cells after 24 hours recovery; B. The kinetics of DNA damage repair after 1 Gy carbon-ion irradiation with/without NU7026 treatment, as measured by relative long PCR amplification.

DNA-PKcs is required for the NHEJ pathway of DNA repair, which rejoins DSBs. Since DNA repair capacity is closely linked to radiosensitivity, NU7026, a specific DNA-PKcs inhibitor, could possibly enhance radiosensitivity by interfering with the repair of DSBs mediated by NHEJ. However, no significant loss of DNA repair capacity was detected in both cells treated with 10 µM NU7026 after 1 Gy carbon-ion irradiation. In addition, PLDR did not restore cellular inactivation of DNA-PKcs-inhibited cells as measured by the survival fraction, indicating that DNA-PKcs could affect radiosensitivity via an alternative pathway independent of its role in DNA damage repair (Figure 3).

### DNA-PKcs Inhibition Resulted in Accelerated Telomere Length Reduction after Carbon-ion Irradiation

**Figure 4:**
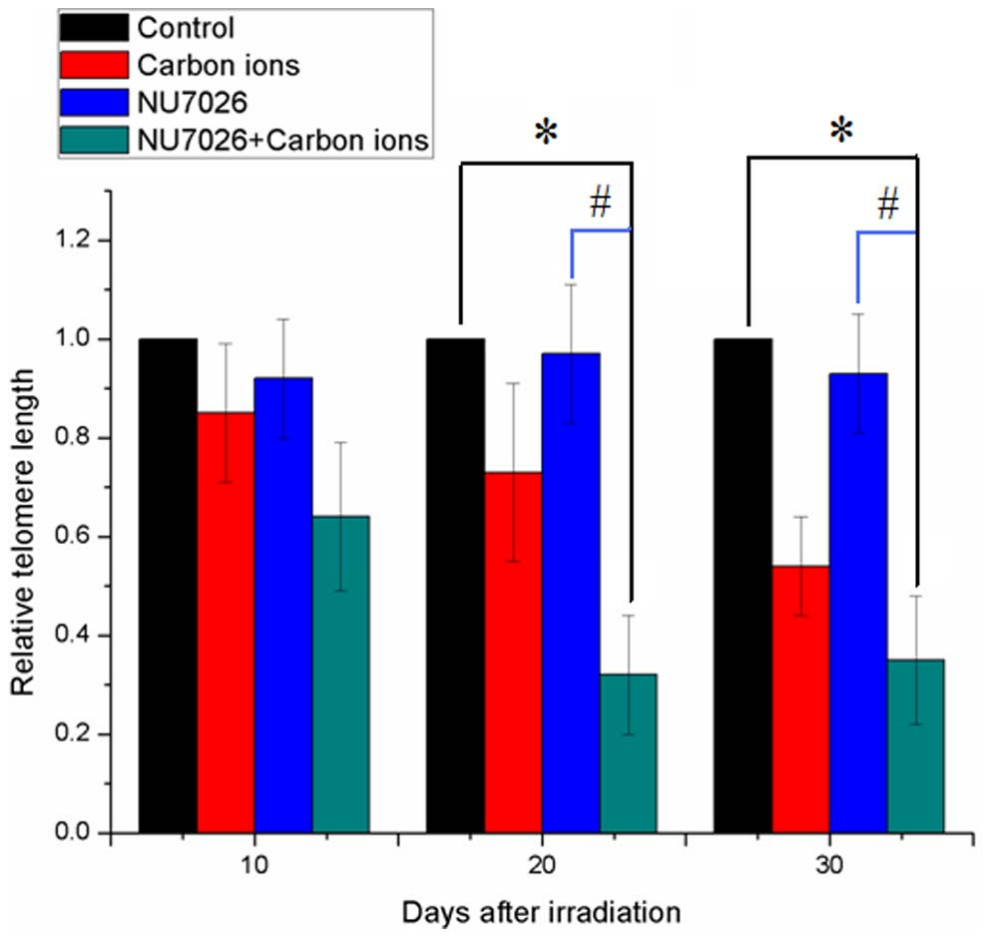
Telomere length reduction in MCF-7 cells with/without NU7026 treatment 30 days after carbon-ion irradiation. MCF-7 cells were incubated with 10 µM NU7026. *: p<0.01 versus control; ^#^: p<0.01 versus 1 Gy carbon-ion irradiation.

Telomere length is a critical factor in the onset of cellular senescence. To investigate whether senescence induced by radiation is due to telomere length reduction, real-time PCR was used to determine the relative quantification of telomere length. As shown in Figure 4, MCF-7 cells cultured with NU7026 exhibited no significant telomere loss; however, telomere length in carbon-ion irradiated cells gradually decreased within 30 days. In particular, telomere length in cells treated with the combination of NU7026 and carbon-ion irradiation decreased more severely. This accelerated telomere loss could account for the increased senescence seen in cells 30 days after irradiation.

### Short Telomeres caused Celluar Inactivation after Carbon-ion Irradiation

**Figure 5:**
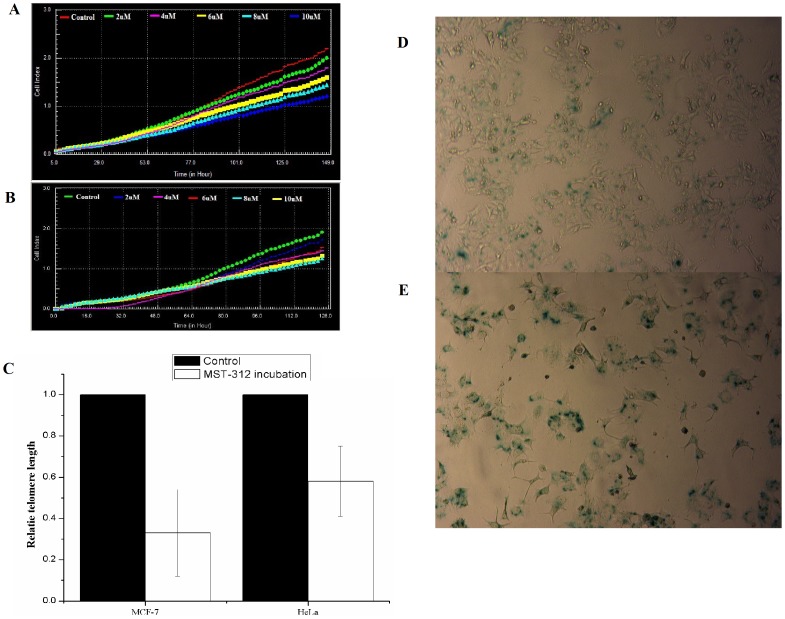
A. Real-time monitoring of adherent cells by the RT-CES System. Real-time monitoring of the growth and proliferation of MCF-7 and HeLa cells in the presence of MST312 using the RT-CES platform. B. Relative telomere length in MCF-7 and HeLa cells after prolonged MST312 incubation. C. Senescence of MCF-7 cells 50 days after MST312 incubation; D. Senescence of MCF-7 cells with shorter telomeres 5 days after 1 Gy carbon-ion irradiation.

To determine whether telomere shortening was the cause of cellular inactivation after carbon-ion irradiation, MCF-7 and HeLa cells with shorter telomeres were established via continuous incubation with the telomerase inhibitor MST312. A cytotoxicity assay showed that 2 µM MST312 did not lead to significant growth arrest compared to the control (Figure 5A–B). Significant shortening of telomere length was detected in MCF-7 cells after 50 days and in HeLa cells after 30 days continuous incubation with MST312 (Figure 5C). (Figure 5B). ß-Galactosidase histochemical staining revealed that while minor senescence was detected in MCF-7 cells after 50 days co-incubation with MST312 (Figure 5C), 1 Gy carbon-ion irradiation induced extensive senescence in these cells (Figure 5D). Carbon-ion radiation induced high level of apoptosis in HeLa cells with shorter telomere (Table 2).

## Discussion

One of the important differences between high-LET and low-LET radiation is that high-LET radiation produces more densely distributed and clusters of DSBs than low-LET radiation. It has been suggested that it would be more challenging for the cellular DNA repair system to repair clustered DNA damage [12], [13]. There are two principal DSB repair mechanisms in mammalian cells: NHEJ and homologous recombination (HR). It is believed that NHEJ is the dominant DSB repair pathway in mammalian cells [14]–[17]. However, short DNA fragments induced by heavy-ion irradiation were reported to inhibit the NHEJ process [18]. Moreover, heavy-ion irradiation induced complex DSBs which are thought to be repaired by HR [19]. Thus, the additional inhibitory effect of NHEJ could have imposed a limited effect on the repair of carbon-ion irradiation-induced DNA damage in our experiment. The inhibition of DNA-PKcs slowed the kinetics of DNA repair in our study and may have been due to the slower DNA repair kinetics of HR, compared to the NHEJ process. As DNA damage was eventually repaired within 24 hours after irradiation, the enhanced radiosensitivity could have been caused by other factors, such as critical telomere shortening. Drissi et.at. reported that short telomeres induce chromatin structure changes that limit access of activated ATM to its downstream targets on the chromatin, which may account for the increased radiation sensitivity seen with telomere shortening [20]. The irrelevance of NHEJ repair to radiosensitivity was also evident in our PLDR data, which demonstrated that even though most of the DNA damage was repaired after 24 hours incubation, DNA-PKcs inhibition could still lead to lower cell survival.

Sabatino et.al suggested that telomere shorten after irradiation may represent an important mediator between radiation exposure, ROS formation and vascular damage [21]. The marked increase in radiosensitivity following DNA-PKcs inhibition could have been caused by the other role of DNA-PKcs, which acts as a telomere binding protein [22]. DNA-PKcs abrogation may lead to a faster rate of telomere degradation due to its interaction with telomerase in telomere maintenance [23]. Studies have shown that DNA-PKcs was also involved in telomere end protection. The characteristics of telomere dysfunction with DNA-PKcs inhibition has been extensively studied by Bailey SM et.al. [24], [25]. These authors demonstrated that uncapped telomeres with DNA-PKcs are inappropriately detected and processed as DSBs, thus participating in ionizing radiation-induced telomere-DSB fusion events.

As a critical component in telomere end-capping [26], DNA-PKcs inhibition may disturb the correct access of telomerase to telomeres, ultimately resulting in telomere shortening. Since most cancer cells harbor shorter telomeres than normal cells, their telomeres are more likely to be reduced to a critical length if telomerase is dysfunctional. In the present study, accelerated telomere shortening was detected in DNA-PKcs-inhibited cells after carbon-ion irradiation in MCF-7 cells, accompanied by extensive cellular senescence. We cannot detect telomere shortening in HeLa cells after carbon-ion with DNA-PKcs inhibition, due to their incapacity for continuous proliferation. The significant apoptosis in HeLa cell could be mediated by DNA damage response. However, DNA damge assay revealed that DNA damage was efficiently repaired within 24 hours after irradiation. Since DNA-PKcs not only participated in DNA double stand repair, but also act as a crucial element for telomere end capping, it’s therefore possible that DNA damage response was induced by dysfunctional telomere. With the establishment of HeLa and MCF-7 cells harbored shorter telomere, we confirmed that telomere dysfunction could contribute to cellular inactivation of cancer cells after carbon-ion irradiation. These results provide evidence that DNA-PKcs inhibition may result in radiosensitization due to its critical role in telomere end capping.

In conclusion, we found that NU7026 significantly sensitized MCF-7 and HeLa cells to carbon-ion irradiation. This increased radiosensitivity was unlikely caused by incomplete DNA damage repair, but by telomere dysfunction. Our findings provide evidence to suggest that targeting the telomere end-capping protein could be an effective way of treating breast cancer.
